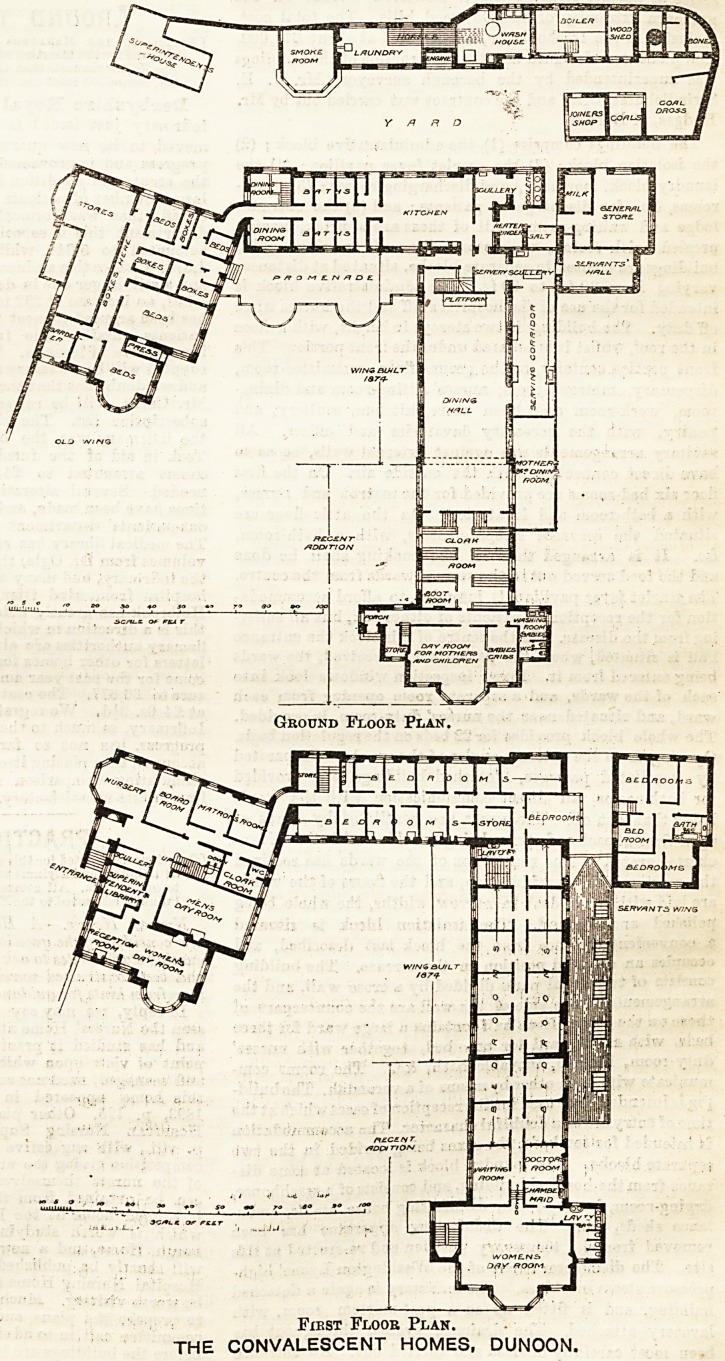# Hospital Construction

**Published:** 1896-01-25

**Authors:** 


### The Institutional Workshop.

### HOSPITAL CONSTRUCTION.

### THE CONVALESCENT HOMES, DUNOON.

The latest addition to these buildings gives about fifty new beds, and, with the accommodation previously provided and a proportional enlargement of the dining hall, provides for the reception of 250 patients. Other improvements dictated by experience are the planning of a separate day-room and a separate dining-room (conveniently placed with regard to the general serving-room) for mothers with young children, and a crib-room for babies leading out of the mothers' day-room. A large cloak-room and boot-room supply lockers for each patient to store his or her valuables, and a convenient place for changing outdoor garments. These are excellently arranged on the ground floor, near the entrance to the new building, and connected with the dining hall. The floor above provides a pleasant women's day-room and lavatory accommodation, and some of the additional bedrooms, with a doctor's-room and waiting-room over part of the cloak-room. The central corridor appears to be lighted principally from, and to derive fresh air through, the bedrooms, as the building is carried up another floor above that shown on the plan here reproduced. This is an objectionable arrangement in any building, but especially so in one intended for convalescent individuals unknown to one another. Another apparent want in the plan is a proper supply of bath-rooms for the very large number of patients to be accommodated. Thus, on the first floor there are 25 bed-rooms without a single bath-room available for the occupants, except by going down to the floor below, where half-a-dozen bath-rooms are shown adjoining the kitchen. The home being for both sexes such an arrangement is practically open to grave difficulties. The plans have, however, many points to commend them, especially in regard to the supply of accommodation essential to such establishments, but too often neglected. The architects are Messrs. Salmon and Son, of Glasgow.

GROUND FLOOR PLAN.

FIRST FLOOR PLAN.

THE CONVALESCENT HOMES, DUNOON.

## Figures and Tables

**Figure f1:**